# *Chlamydia trachomatis *diversity viewed as a tissue-specific coevolutionary arms race

**DOI:** 10.1186/gb-2008-9-10-r153

**Published:** 2008-10-23

**Authors:** Alexandra Nunes, Paulo J Nogueira, Maria J Borrego, João P Gomes

**Affiliations:** 1 Department of Infectious Diseases, National Institute of Health, Av. Padre Cruz, 1649-016 Lisbon, Portugal; 2 Department of Epidemiology, National Institute of Health, Av. Padre Cruz, 1649-016 Lisbon, Portugal

## Abstract

**Background:**

The genomes of pathogens are thought to have evolved under selective pressure provided by the host in a coevolutionary arms race (the 'Red Queen's Hypothesis'). Traditionally, adaptation by pathogens is thought to rely not on whole chromosome dynamics but on gain/loss of specific genes, yielding differential abilities to infect distinct tissues. Thus, it is not known whether distinct host organs differently shape the genome of the same pathogen. We tested this hypothesis using *Chlamydia trachomatis *as model species, looking at 15 serovars that infect different organs: eyes, genitalia and lymph nodes.

**Results:**

We analyzed over 51,000 base pairs from all serovars using various phylogenetic approaches and a non-phylogenetic indel-based algorithm to study the evolution of individual and concatenated loci. This survey comprised about 33% of all single nucleotide polymorphisms in *C. trachomatis *chromosomes. We present a model in which genome evolution indeed correlates with the cell type (epithelial versus lymph cells) and organ (eyes versus genitalia) that a serovar infects, illustrating an adaptation to physiologically distinct niches, and discarding genetic drift as the dominant evolutionary driving force. We show that radiation of serovars occurred primarily by accumulation of single nucleotide polymorphisms in intergenomic regions, housekeeping genes, and genes encoding hypothetical and cell envelope proteins. Furthermore, serovar evolution also correlates with ecological success, as the two most successful serovars showed a parallel evolution.

**Conclusion:**

We identified a single nucleotide polymorphism-based tissue-specific arms race for strains in the same species, reflecting global chromosomal dynamics. Studying such tissue-specific arms race scenarios is crucial for understanding pathogen-host interactions during the course of infectious diseases, in order to dissect pathogen biology and develop preventive and therapeutic strategies.

## Abstract 2

Analysis of 15 serovars of *Chlamydia trachomatis* reveals an evolutionary arms race in pathogen-host interactions.

## Background

When two species interact with each other, such as a pathogen and human, a never-ending reciprocal and dynamic adaptation process takes place. Whereas the 'goal' of the human being is to try to avoid, solve or minimize the infection, the 'goal' of the pathogen is to deal with this constant host environmental and immune pressure, through genomic evolutionary changes, in order to win this arms race [1-4]. Typically, genome evolution within same-species strains of a pathogen has been studied mainly in the light of horizontal gene transfer (HGT) at specific chromosome loci [5,6], as for *Escherichia coli *[7,8], *Staphylococcus aureus *[9], *Streptococcus pyogenes *[9], *Salmonella enterica *[10], *Shigella flexneri *[11], and *Pseudomonas syringae *[3]. An extreme example is provided by the well-studied *E. coli*, where strains K-12 and O157 differ by more than 1 million base pairs [12], and same-serovar strains were found to present profound differences in gene content [13,14]. Globally, these targeted HGT events reflect different pathoadaptation processes for microrganisms with reversible genome size-plasticity; depending on the transitory 'cassette-genes' carried at any specific time, the pathogenecity or ability of these microrganisms to infect different tissues may vary [7]. Thus, generally, these processes rely on gain/loss of virulence/colonization factors rather than reflect whole chromosomal dynamics, the evaluation of which remains complex. Indeed, assessment of tissue-specific adaptive evolution at the whole genome level demands that same-species strains of a pathogen specifically and non-transitorily infect different tissues. Therefore, on behalf of the arms race theory assumed by the evolutionary Red Queen's Hypothesis [15,16], one question arises: do distinct host organs differently shape the genome of the same pathogen? No microrganism is more suitable than *Chlamydia trachomatis*, the most prevalent sexually transmitted bacterial pathogen worldwide, to test this hypothesis, as the species comprises several serovars with a wide range of specific human tissue tropism. This pathogen is mainly classified into 15 serovars based on the differential immunoreactivity of the major outer membrane protein (MOMP), constituting three disease groups [17]: serovars A-C and Ba are commonly associated with ocular trachoma; serovars D-K infect the epithelial cells of genitalia and are normally found in non-invasive sexually transmitted infections (where serovar E represents about one-third of all infections, and together with serovar F constitute up to 50% of them); serovars L1-L3 are also sexually transmitted but are invasive and disseminate into the local lymph nodes causing lymphogranuloma venereum (LGV). However, in the context of this classification system, the evaluation of adaptive evolution becomes enigmatic because there is no correlation between it and *C. trachomatis *tropism nor with the ecological success of the different serovars, as strains with different organ specificities are placed within the same classification group.

As occurred for *Mycobacterium leprae *[18], *Rickettsia prowazekii *[19], and the aphid endosymbiont *Buchnera aphidicola *[20], the first stages of *Chlamydia *evolution consisted of a massive genome reduction upon becoming an obligate intracellular parasite [21,22]. However, comparative genomics over the few currently fully sequenced *C. trachomatis *genomes [20,23-25] revealed that gene decay is not involved in the more recent evolutionary stages. Indeed, contrary to most pathogens, the core- and the pan-genome [6] of this microrganism are near identical, indicating that the factors involved in the differential organ specificity among serovars are not acquired by gene transfer [24].

To evaluate if distinct arms races occur between different infected human organs and this pathogen's serovars, we performed high-scale concatenation-based phylogenomics, using about one-third of all chromosome single nucleotide polymorphisms (SNPs). So far, in contrast to the ocular group, only one strain from the epithelial-genital and LGV groups has been fully sequenced [20,23-25], making our multiple-loci scrutiny of all 15 serovars the ideal tool to track the evolutionary diversity of a microrganism characterized for its distinct infection niches. Here, we show a matchless model of SNP-based adaptive evolution of same-species strains to each infected cell-type and organ that relies on whole chromosome evolutionary dynamics, unlike previous reports for other pathogens focused on specific gene gain/loss.

## Results

### Evaluation of the degree of polymorphism for the selected loci

**Figure 1:**
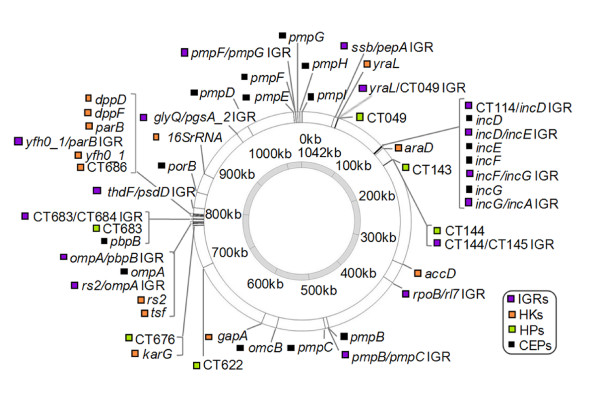
Loci distribution in the approximately 1.04 Mb *C. trachomatis *circular chromosome. Gene names and open reading frame numbers are based on the *C. trachomatis *D/UW3 genome annotation [GenBank: AE001273]. Loci categories are illustrated by different colors. Only the first nucleotide of each locus is marked on the figure.

Considering that the strain radiation yielding the present-day chlamydial serovars likely occurred over millions of years [26], the use of reference strains is an accurate strategy as they were isolated only a few decades ago. Thus, in this evolutionary survey, we used the traditional reference strains that represent all 15 *C. trachomatis *serovars. We selected 51 polymorphic loci (approximately 51,000 bp) dispersed throughout the chromosome (Figure 1; Additional data file 1) that represent the following loci categories: 16 intergenomic regions (IGRs); 16 genes encoding cell envelope proteins (CEPs); 13 housekeeping genes (HKs); and 6 genes encoding hypothetical or unclassified proteins (HPs) (Additional data file 2). In order to evaluate the degree of polymorphism of these loci in comparison with the whole chromosome, we used the data generated from two of the five fully sequenced genomes, A/Har13 (ocular) [23] and D/UW3 (epithelial-genital) [21]. We observed in the studied 51 loci a global mutation rate 14.3-fold higher than in the remaining chromosome regions (Fisher's exact test, *P *< 0.001). Moreover, we found 1,099 SNPs in these 51 loci between A/Har13 and D/UW3, which is greater than 200-fold more than what has been studied to date through concatenation [27], and comprises about 33% of the whole chromosome SNPs, indicating that our results could be scaled up to the full-chromosome level.

Additionally, a global overview of GC content revealed a mean value for all loci categories (data not shown) that is similar to the total mean GC content of approximately 41% observed for the fully sequenced genomes [21,23-25] with a standard deviation of 2.9%, which is not indicative of any putative HGT event.

### Correlation of individual loci with tissue-specific strain radiation

**Figure 2:**
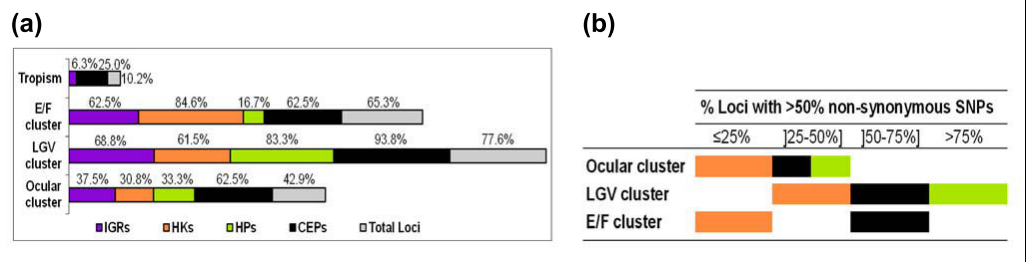
Phylogenomics of individual loci versus strain segregation. **(a) **Phylogenetic strain segregation. Loci categories are illustrated by different colors. Numbers on the top of each bar show the percentage of loci, within each category, that generate a tree where a full tissue tropism, or a particular cluster of strains, or an E/F co-segregation is observed. **(b) **Percentage of loci (within each functional category) for which the majority of SNPs yield an amino acid change. The color scheme for the represented loci categories is the same as (a).

We used phylogenomics to correlate each individual locus with tissue-specific strain radiation. Only four (25.0%) CEPs (*incD*, *incE*, *pmpF *and *pmpH*) and one (6.3%) IGR (*incD*/*incE*) comprehensively grouped the strains according to their cell-type/organ appetence (that is, revealed a larger evolutionary distance between strains with different niche appetencies than between strains infecting the same niche; Figure 2a). This clustering seems to be associated with loci revealing a higher p-distance-based polymorphism (Mann-Whitney *P *= 0.025). A full segregation by cell-type/organ appetence was not seen for most of the remaining CEPs due to the heterogeneity among the genital strains, where serovars E and F frequently form a separate cluster for 62.5% of CEPs (Figure 2a). Globally, 77.6% of loci belonging to different functional categories grouped strains that invade the lymph nodes as an individual cluster (LGV cluster), and the clustering of strains infecting the ocular tissue (ocular cluster) was also frequent. As above, we identified a significant association between a higher absolute number of SNPs and both the occurrence of a LGV cluster and an ocular cluster for each locus (Mann-Whitney *P *= 0.037 and *P *= 0.045, respectively). Interestingly, from the loci that better illustrate adaptation to lymph nodes, 80% of HPs and 53% of CEPs, compared with only 29% of HKs, show >50% non-synonymous SNPs (Figure 2b). Considering the DNA replication process, all SNPs on one strand that may imply strain segregation will also have the same impact on the other DNA strand. However, from the 51 loci that we used, only 4 pairs of loci overlap and the overlapping region never exceeds 10 bp (data not shown), which makes this effect negligible. Overall, these results suggest that the distinct genetic variability of strains infecting a specific cell-type/organ likely reflects an evolutionary adaptation process.

**Figure 3:**
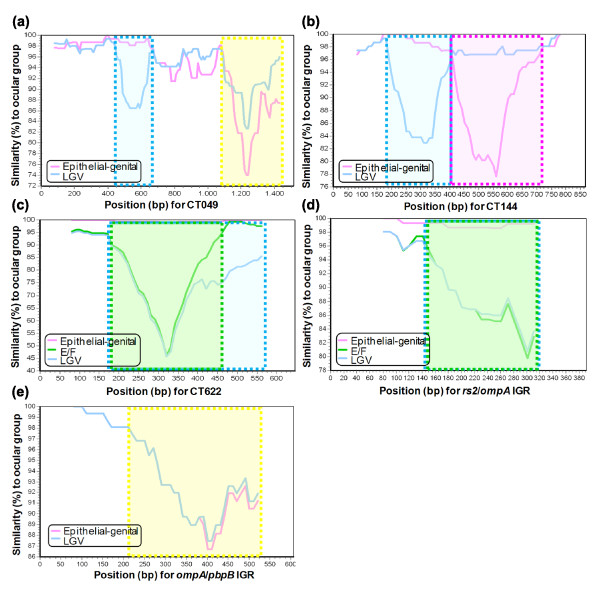
Identification of loci domains characteristic of strains infecting a specific biological niche. SimPlot graphs show the nucleotide similarity between the ocular, epithelial-genital and LGV strains for **(a) **CT049, **(b) **CT144, **(c) **CT622, **(d) ***rs2/ompA *IGR and **(e) ***ompA/pbpB *IGR. Epithelial-genital (pink) and LGV (blue) strains are compared to the ocular strains (represented in the upper x-axis). For CT622 (c) and *rs2/ompA *IGR (d), where an E/F clustering apart from the other epithelial-genital strains was observed, SimPlot analysis has also involved serovars E/F (green). For each panel, the loci domains that are specific to LGV, epithelial-genital, ocular or E/F strains are bordered by boxes in blue, pink, yellow and green, respectively. For panels (c) and (d), LGV and E/F specific domains partially or completely overlap, respectively. The represented domains correspond to a non-random fixation of SNPs, yielding clusters of amino acid changes.

By performing intra-locus analysis, we observed that three HPs (CT049, CT144 and CT622) and two IGRs (*rs2*/*ompA *and *ompA*/*pbpB*) revealed distinct domains in which SNPs are concentrated, instead of being randomly distributed, and are associated with strains that infect a specific cell-type/organ (Figure 3). For these HPs, the SNP domains correspond to clusters of amino acid changes in the protein sequence (data not shown), mirroring the previous findings for some polymorphic membrane protein genes [28]. Unfortunately, there is no assigned role for these open reading frames, which rules out any speculation about the functional implications of these specific clustered amino acid alterations. Nevertheless, this tissue-specific amino acid clustering points to a targeted fixation of mutations that may reflect the host-pathogen specific interaction within each organ.

### Genomic analysis of the concatenated loci

We evaluated the nucleotide sequence variation in each concatenated loci category (Table 1). We highlight the multi-loci concatenation approach as a powerful tool to generate robust phylogenomic inferences, even when individual loci have evolved with different substitution patterns [29-31]. Overall, the HPs exhibit the highest number of variable sites (10.3%), whereas the HKs are the least variable (3.3%), which is supported by the mean p-distance values. Curiously, the IGRs show polymorphism similar to the CEPs. Globally, concatenation of all 51 loci yielded a 'super' sequence of up to 51,074 bp for each of the 15 reference strains, showing a mean of 1,032.1 (standard error (SE) 17.2) nucleotide differences.

**Table 1:** Genetic polymorphism for the concatenated sequences

	Loci categories				
		
	IGRs	HKs	HPs	CEPs	Total
Size	3,834	7,984	3,906	35,350	51,074
Variable sites*	192 (5.0%)	267 (3.3%)	402 (10.3%)	1800 (5.1%)	2,662 (5.2%)
Parsimony informative sites^†^	147 (76.6%)	233 (87.3%)	372 (92.5%)	1,656 (92.0%)	2,408 (90.5%)
Overall mean distance (nucleotides)	65.8 (SE 4.7)	96.4 (SE 5.3)	168.1 (SE 7.6)	701.9 (SE 15.6)	1,032.1 (SE 17.2)
Overall mean p-distance (nucleotides)	0.0172 (SE 0.0013)	0.0121 (SE 0.0007)	0.0430 (SE 0.0020)	0.0199 (SE 0.0004)	0.0202 (SE 0.0003)

Calculations based on the alignment of the 15 strains. *The percent value is relative to the respective size of each loci category. ^†^The percent value is relative to the respective number of variable sites of each loci category.

### Evolutionary history of *C. trachomatis*

**Figure 4:**
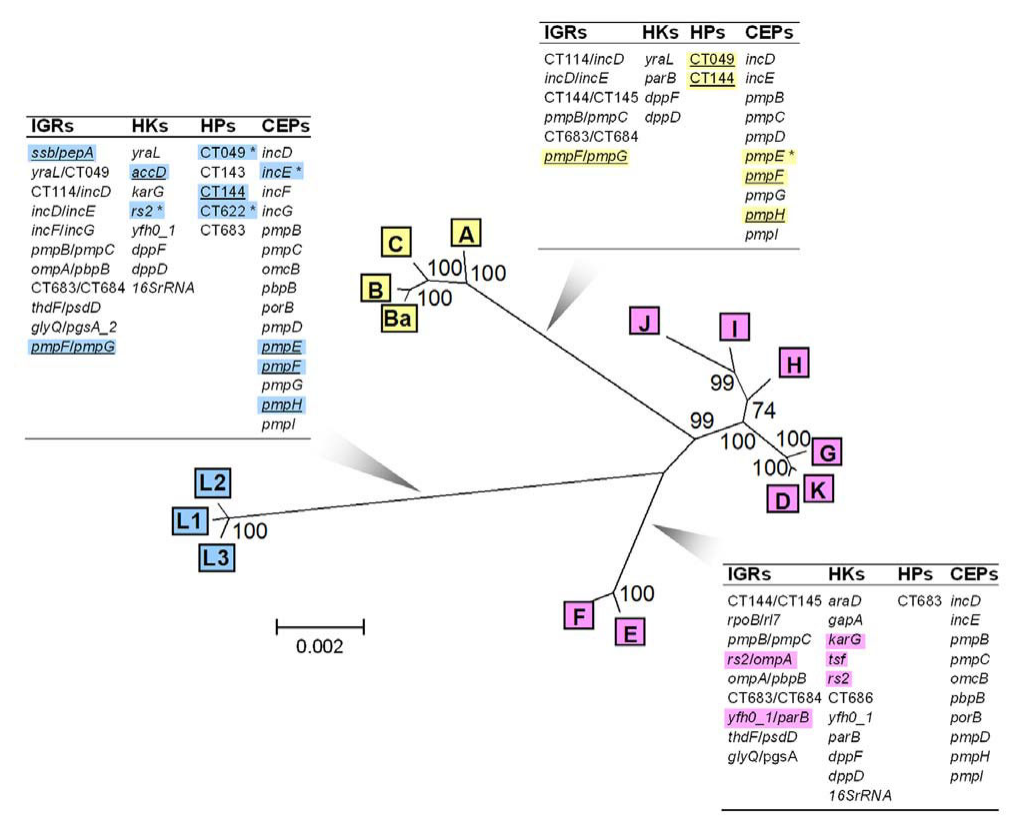
*C. trachomatis*'s evolutionary history. The global phylogenetic tree (NJ, K2P model) is based on about 50,000 bp/taxa. Bootstrap values (1,000 replicates) are shown next to the branch nodes. Ocular, epithelial-genital and LGV strains are represented within yellow, pink and blue boxes, respectively. Charts show the loci contributing to taxa segregation for the assigned tree branches, where the most prominent ones (genetic variability >4%) are highlighted with the corresponding color. Within these highlighted loci, the ones revealing polymorphism (defined as ≥10 SNPs, or >50% amino acid changes when <10 SNPs) among strains infecting the same organ (eyes or lymph nodes), may be involved in pathogenesis (marked with asterisks). Loci without polymorphism within strains infecting the same organ likely reveal the final stages of adaptive evolution (underlined).

Due to the speed and efficiency of the neighbor joining (NJ) method in inferring large phylogenies [32,33], we used this approach on concatenated data. The NJ phylogenies inferred from the four concatenated loci categories (Additional data file 3) are consistent with most of the respective individual loci trees. Although only the CEP category clearly segregates strains by the disease they cause, the other categories show a notable segregation of at least one disease group, suggesting that heterogeneous loci categories are involved in the arms race process. The global phylogenetic tree presented in Figure 4 (where each taxon is represented by about 50,000 bp) reveals the putative final picture of *C. trachomatis*'s evolution, showing strain grouping according to the cell-type (epithelial and lymph cells) and organ (eyes and genitalia) that they infect. These distinct segregations are supported by maximum bootstrap values (99-100%) in the nodes that separate disease groups, reinforcing that the targeted and distinct fixation of nucleotide changes on strains infecting a specific cell-type/organ are likely adaptive and barely the consequence of genetic drift. In fact, the genetic distance matrix (Table 2) shows that all strains that preferentially infect the eyes revealed only 0.27% (SE 0.02%) differences among them, but shows a mean genetic distance 7.4- and 11.2-fold higher (corresponding to 983 (SE 20) and 1,484 (SE 42) nucleotides) to strains infecting the epithelial-genital and lymph node tissues, respectively. Also, the LGV strains differ by only 69 (SE 8) nucleotides, whereas their distance to the epithelial-genital strains is 1,226 (SE 34) nucleotides. A separate main branch involving all epithelial-genital strains was not comprehensively seen for any individual loci (except for the CEPs *pmpF *and *pmpH*; data not shown) due to the separation of E and F strains. Indeed, the latter has a mean genetic distance of 673 (SE 16) nucleotides to the other epithelial-genital strains (Table 2). Similar NJ tree topologies were obtained for the three models used to estimate evolutionary distances (Kimura 2-parameter (K2P), Jukes-Cantor or Tamura-Nei) as well as for the maximum parsimony method (data not shown), with only slight variations in the bootstrap values, which supports the robustness of these distinct arms race scenarios.

**Table 2:** Overall mean genetic distances within and between disease groups

	Nucleotide differences*	Genetic distance (%)*
**Within-group means**		
Ocular	133 (SE 10)	0.27 (SE 0.02)
Genital (without E/F)	309 (SE 14)	0.63 (SE 0.03)
Genital (with E/F)	460 (SE 13)	0.93 (SE 0.03)
LGV	69 (SE 8)	0.14 (SE 0.02)
		
**Between-group means**		
Ocular/Genital (without E/F)	942 (SE 21)	1.91 (SE 0.04)
Ocular/Genital (with E/F)	983 (SE 20)	2.00 (SE 0.04)
Ocular/LGV	1,484 (SE 42)	3.02 (SE 0.08)
LGV/Genital (without E/F)	1,241 (SE 36)	2.52 (SE 0.07)
LGV/Genital (with E/F)	1,226 (SE 34)	2.49 (SE 0.07)
Genital/E-F	673 (SE 16)	1.37 (SE 0.03)

*Genetic distances were estimated in the concatenated approximately 50,000 bp/taxa.

We also highlight the loci that most contribute to the final tree topology (Figure 4), as they may be relevant for the evolutionary adaptation to each specific niche. Among these loci, we have found either highly conserved or polymorphic loci for strains infecting the same cell-type/organ. The former may represent a step forward in the evolutionary process by revealing the final stages [1] of this tissue-specific adaptive evolution, while the latter may also be involved in pathogenic differences between strains infecting the same tissue [25]. The most extreme case is given by the CEP *pmpF*, where all the strains that infect the lymph nodes are 100% similar but show a mean distance of 312 and 421 SNPs to strains infecting the epithelial-genital and ocular tissues, respectively. In contrast, the epithelial-genital strains reveal up to 129 SNPs among them (data not shown). Although less markedly, CT049 is polymorphic among the LGV strains but near 100% identical among the ocular strains.

Additionally, we identified loci that do not seem to have influenced adaptation to each niche, since they generate an incongruent strain-radiation (Table 3), and whose polymorphism may thus be a consequence of genetic drift. However, previous results have demonstrated the involvement of some of these loci (CT622, *tsf*, *rs2 *and *pbpB*) in the pathogenesis of trachoma [25]. As expected because of the serovar multiplicity, the epithelial-genital group revealed a higher number of polymorphic loci, and, overall, these loci belong to different categories. In contrast, strains infecting the lymph nodes constitute the most homogeneous group.

**Table 3:** Polymorphic loci among strains that infect the same biological niche

Ocular	Epithelial-genital	LGV
IGR (*rs2*/*ompA*)	IGR (*rs2*/*ompA*)	*tsf*
IGR (*ompA*/*pbpB*)	*karG*	*ompA*
*tsf*	*rs2*	
*ompA*	CT049	
*pbpB*	CT144	
	CT622	
	*pmpC*	
	*ompA*	
	*pmpE*	

The represented loci may hypothetically be involved in the pathogenesis of ocular trachoma, genital infections or LGV disease, but do not contribute to the adaptive evolution of strains to each correspondent biological niche (Figure 4). Polymorphism was defined as >4% nucleotide differences or >40 SNPs (for genes >3 Kb).

### Impact of small insertions/deletions (indels) on tissue-specific strain radiation

In order to have a more complete picture of the evolution of the serovars, we studied the chromosomal occurrence of small insertion/deletion (indel) events, which are non-phylogenetic parameters. We observed 84 small indel events (from 1-43 bp) inside the global concatenated loci for all strains, which mainly occurred within the IGR and CEP categories (Additional data file 4). None of these events was found to disrupt the coding sequence of the respective loci, indicating the absence of gene decay in the studied regions.

**Figure 5:**
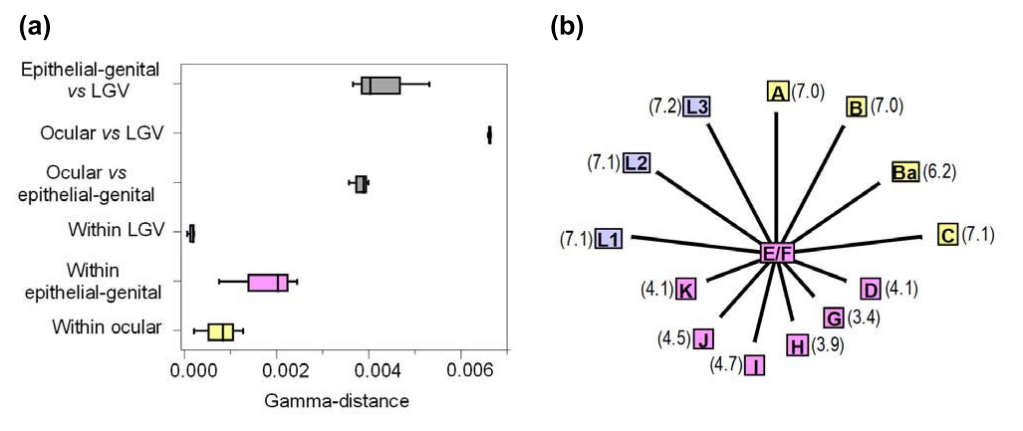
Impact of indel events on *C. trachomatis*'s evolution and ecological success. **(a) **Evolutionary γ-distances for the global concatenated data within (colored boxes) and between (grey boxes) disease groups (ocular, epithelial-genital and LGV). Boxes represent the variability of all distance estimates, while the vertical line within each box divides 50% of all values. The minimum and maximum distances are represented by the extremes of each horizontal line. **(b) **Impact of indel events on *C. trachomatis*'s ecological success. The mean γ-distances from any ocular (yellow), epithelial-genital (pink) or LGV (blue) strain to E/F strains are represented in parentheses. Each evolutionary distance was normalized against the distance between E and F. The relative length of each line is represented in the correct scale.

For the global concatenated data, we estimated the evolutionary distances using the indel-based parameter γ [34], which computes the number of gap nucleotides per nucleotide site between those sequences, while SNPs are not considered. The γ-distances (Figure 5a) are highly concordant with phylogenomic analyses, showing high heterogeneity within the epithelial-genital strains, and remarkable homogeneity among the LGV strains. Also, they revealed a segregation of strains by their cell-type/organ appetencies, which supports the tissue-specific arms race scenario.

### Evolutionary inferences on the ecological success

Analysis of the global phylogenetic tree (Figure 4) also shows that the two most prevalent genital serovars worldwide, E and F, are closely related and separated from the other epithelial-genital strains. This segregation is observed for the majority of loci, with the exception of the HPs (Figure 2a). From all these loci, 70% of CEPs show an amino acid replacement for >50% of SNPs, compared to only 20% of HKs (Figure 2b). Curiously, the most remarkable segregation of E and F was seen for two IGRs (*rs2*/*ompA *and *yfh0_1*/*parB*) and three HKs (*karG*, *tsf *and *rs2*) (Figure 4). Furthermore, for the still unclassified protein gene CT622 and for the IGR *rs2/ompA*, we observed a non-random distribution of SNPs that are present in serovars E and F but not in the other epithelial-genital strains (Figure 3c,d). Finally, the mean γ-distance from any epithelial-genital strain to serovar E or F was from 3.4-fold (between G and E/F) to 4.7-fold (between I and E/F) higher than the distance between E and F (Figure 5b), which supports this close relationship between the two most ecologically successful serovars.

## Discussion

We have hypothesized that distinct arms races may occur inside the same host when the same pathogen is able to infect different organs. In contrast to free living bacteria, where HGT is strongly associated with a pathogen's adaptive evolution [3,5-11], *Chlamydia *has been characterized by genetic isolation and, while cumulative studies suggest that HGT has almost certainly occurred in Chlamydiaceae [35-37], there is no report to date of transferable mobile elements in *C. trachomatis*. Here, we demonstrate that *C. trachomatis *strains that preferentially infect the eyes, the epithelial-genital cells or the lymph nodes present a distinct evolutionary pattern likely illustrating a SNP-based tissue-specific arms race.

In order to develop a more compelling argument for a causal link between genome profile and cell/organ appetence, the use of genetic modification and especially the use of animal models are appealing approaches. However, *C. trachomatis *is genetically non-tractable and, except for the cynomolgus monkey (accurate for studying the trachoma pathology) [25], no suitable animal model exists for the three types of *C. trachomatis *disease. Also, there is no *in vitro *model, such as cell culture, that mirrors the chlamydial infection *in vivo*, and it has been previously demonstrated that intensive serial passaging of chlamydial strains yielded no mutations on the most variable chlamydial gene (*ompA*) [38]. Furthermore, it would be inconceivable that these approaches could represent millions of years of chlamydial evolution.

It is believed that the LGV biovar was the first to diverge from a common *C. trachomatis *ancestor when new primate hosts evolved after the dinosaur extinction, whereas separation of genital and ocular serovars might have occurred with the appearance of early humanoid primate hosts [26]. The skill to colonize different organs and cell-types likely developed through indel events and SNP accumulation on virulence/colonization factors. So far, chlamydial putative virulence factors, such as the type III effector *tarp *[23], the cytotoxin gene [39], and especially the tryptophan operon [40,41], are the best candidates for providing that skill. In particular, while the first of these factors differentiates the LGV strains from the other groups, the other two differentiate the strains colonizing the genitalia from the strains colonizing other niches. For example, it was clearly demonstrated that only strains possessing a functional *trpBA *operon are able to colonize the genital tract [41]. With respect to type III effectors, although their role in *C. trachomatis *tropism is not clear, it was shown that evolutionary genetic diversification of the type III effector *HopZ *family, via horizontal transfer, had clear implications for *Pseudomonas syringae *host specificity [3]. However, none of the chlamydial putative virulence factors fully explain the existence of the three major tropism groups made up from the different serovars. Also, the putative emergence of tissue-specific adhesins cannot be discarded.

With regard to our results (Figure 4), strain radiation within each disease group likely occurred because of accumulation of mutations throughout the chromosome caused by environmental and immune pressure in each niche, giving rise to the contemporary serovars. Within the genitalia, the higher serovar multiplicity and radiation of epithelial-genital strains compared to the LGV strains would be unexpected in the light of the earlier evolutionary divergence of the latter [26]. However, besides the different host immune responses in those niches, the epithelial-genitalia environment presents pH and hormonal fluctuations that are variable among individuals, and also an abundant nutrient-competing flora, which could have strongly influenced the evolutionary pathway of the infecting strains. In support of this, nutrient-competing flora were shown to be a major factor in the successful pathoadaptation of *Salmonella enterica *serovar Typhimurium to the intestinal tract, as the inflammatory process induced by this pathogen was shown to make a negative impact on mainly the other colonizing microrganisms and, thus, a positive impact on its arms race with the host [42].

Globally, we have observed that the loci that most contribute to strain segregation by cell-type/organ are spread throughout the chromosome (Figure 1) and belong to different functional categories, suggesting that this dynamic evolutionary adaptation is a general trait of the entire genome. Whereas the contribution of CEPs is likely associated with putative structural, antigenic or host-adhesion roles, no assumption can be made for the HPs. However, we found that HPs were the most variable among the serovars, with an overall polymorphism 2.2-fold higher than the CEPs (Table 1), which suggests a higher involvement in chromosomal dynamics. With respect to IGRs, we speculate that their contribution to strain segregation may be associated with recombination events that may promote genetic variability, as we recently described [43]. Nevertheless, the high variability of IGRs was surprising, as they commonly involve regulatory regions that are expected to be conserved; thus, the existence of random genetic drift may also be considered for IGRs. Finally, although the HKs are involved in strain segregation, the vast majority of them showed <50% non-synonymous mutations (Figure 2b), which is consistent with their role in essential biological functions.

It is known that in populations without HGT and with bottlenecks, as is the case for *C. trachomatis*, random genetic drift can play a major role in evolution, being responsible for the fixation of unfavorable mutations [44]. However, our results suggest that chlamydial strain segregation according to tropism properties occurred mainly through an adaptive evolutionary process and not through dominant genetic drift. Several arguments point in this direction: the statistical association found between most polymorphic loci (number of SNPs/loci and p-distance/loci) and the strain clustering according to their tissue specificity; Chlamydiae presents a relatively high ratio of non-synonymous to synonymous changes when compared, for example, to *E. coli *and *Buchnera *[26], further supported by our findings where the majority of HPs and CEPs involved in the segregation of the LGV strains showed >50% non-synonymous SNPs (Figure 2b); for at least eight loci (CT049, CT144, CT622, *pmpE*, *pmpF*, *pmpH*, *rs2/ompA *IGR and *ompA/pbpB *IGR), we observed a non-random fixation of SNPs exclusive of same niche-infecting strains (Figure 3), corresponding to specific clusters of amino acid changes in coding sequences; the extremely robust global phylogenetic tree with maximum bootstrap support (99-100%) in the branch nodes where strains are separated by their cell-type/organ specificity (Figure 4); 20 out of the 22 loci that contribute to the segregation of strains that preferentially infect the eyes are also involved in the segregation of strains that colonize the lymph nodes (Figure 4) by presenting a dissimilar and specific SNP pattern; and finally, the well-known differences in environmental and immune pressure as well as competing flora and physiological specificities between ocular, epithelial-genital and lymph node tissues.

Within all the loci that are more likely to be involved in the adaptive evolution to each specific niche, we have found either highly conserved or polymorphic loci among strains infecting the same cell-type/organ (Figure 4), where the most remarkable examples are *pmpF *and CT049 (see Results). We hypothesize that *pmpF *and CT049 may be good representatives of a final stage of the adaptive evolution to the lymph nodes and the eyes, respectively, considering their extreme conservation among the corresponding strains. On the other hand, these genes may be responsible for pathogenic differences among epithelial-genital and LGV strains, respectively, based on their strong polymorphism among the corresponding strains. While PmpF has been implicated as a potential target for the host immune response, as it contains several putative major histocompatibility epitopes [23], biological information for CT049 is lacking.

Additionally, we found several loci that are polymorphic among strains infecting the same cell-type/organ that seem not to have been involved in the adaptation to each niche, but which may have been involved in the pathogenesis of trachoma, genital infections or LGV disease (Table 3). Indeed, 4 of these loci (CT622, *tsf*, *rs2 *and *pbpB*) belong to a pool of 22 genes that are responsible for profound differences in virulence among two *C. trachomatis *ocular strains in nonhuman primates [25].

Interestingly, we also observed a clear evolutionary co-segregation of the two most ecologically successful serovars (E and F). This is intriguing as there is a 15% difference between them in the gene coding for the major antigen (the major outer membrane protein (MOMP)), which constitutes about 60% of the membrane dry-weight [45] and is a putative cytoadhesin [46]. Although it is not known why serovars E and F are the most prevalent worldwide, their ecological success seems not to be associated with intracellular multiplication rate [47], indicating that it is likely defined at the host cell adhesion and entry steps. However, the existence of E/F specific virulence factors or adhesins cannot be addressed in this study. Even so, *tarp *is the unique virulence factor that distinguishes serovar E from the other epithelial-genital serovars (including F), as it presents fewer repeat motifs in the 5' region [23], but its phenotypic consequences are not known. Moreover, a more successful host immune evasion could also be speculated for serovars E and F considering the well-known different antigenic profile among epithelial-genital serovars [48].

Regarding the loci that most markedly contribute to the segregation of serovars E and F, we highlight the IGRs *tsf*, *rs2 *and *rs2*/*ompA *(Figure 4). The first two of these may be involved in hypothetical differences in strain growth [25], while the last involves the regulatory region of *rs2*. This IGR includes specific domains where most SNPs are exclusive of strains E and F (Figure 3d), suggesting a potential impact on the *rs2 *regulation and, thus, on strain growth. Also, the IGR *rs2*/*ompA *is a recombination hotspot for the generation of mosaic structures within chlamydial strains [43], suggesting that recombination may contribute to the ecological success of the two serovars. However, as most SNPs of the CEPs involved in the E/F segregation confer amino acid replacements (Figure 2b), we suggest that the positive selection for the membrane proteins may also be a driving force for the E/F evolutionary divergence, likely through antigenic variability.

## Conclusion

It is not surprising that bacterial populations that evolved in different ecological niches have different profiles of genetic variability. However, contrary to all previous reports for other pathogens focused on HGT events and gene decay, we present evidence of SNP-based, tissue-specific evolutionary adaptation relying on whole chromosome dynamics, as a consequence of the occurrence of dissimilar arms races between the pathogen and diverse host organs. Answering the proverbial question of 'which came first' (tropism or SNPs), the scenario presented here suggests that while some SNPs, on very few and specific loci, are likely responsible for tropism differences, the vast majority of SNPs throughout the chromosome are a consequence of different tissue tropisms and are expected to be involved in maintaining organ appetence, as per the Red Queen's Hypothesis. Mirroring bacterial virulence [6], we present evidence that a 'one size fits all' approach cannot be applied to adaptive evolution. This phenomenon is illustrated by a pathogen believed to infect 140 million people, where the incidence rate can be as high as 30% among adolescent females [49]. We believe that grasping a pathogen's genetic trends with regard to its interaction with the host will be an essential tool in deciphering the molecular genetic aspects of infectious diseases.

## Materials and methods

### Culture of *C. trachomatis *reference strains

We used the most common reference strains representing the 15 *C. trachomatis *serovars: A/Har13, B/TW5, Ba/Apache2, C/TW3, D/UW3, E/Bour, F/IC-Cal3, G/UW57, H/UW4, I/UW12, J/UW36, K/UW31, L1/440, L2/434 and L3/404. McCoy cell culture of all strains plated in T-25 cm^2 ^flasks was performed as previously described [50]. At 48-72 h post-infection, elementary bodies were harvested, and DNA was extracted using QIAamp^® ^DNA Mini Kit (Qiagen, Valencia, CA, USA) according to the manufacturer's instructions. Serovar confirmation of each reference strain was performed using *ompA *genotyping with BLAST comparison of the available GenBank sequences.

### Selection of loci

A GenBank search was performed to look for genomic regions that had been sequenced for at least one *C. trachomatis *reference strain from each of the three disease groups. Up to 93 loci were found, comprising about 84,000 bp of the chromosome, and involving IGRs, HKs, HPs and CEPs. Only non-constant loci were selected (51 of the 93; Figure 1; Additional data file 2) for sequencing the other reference strains if their sequences were not available yet. Automated sequencing was performed as previously described [28]. The DNA sequence data have been deposited in a public database ([GenBank: EU239694-EU239702], [GenBank:EU239705-EU239712], and [GenBank:EU247618-EU247753]). Primer sequences are given in Additional data file 5. For all strains, five types of concatenated sequences were created in a head-to-tail fashion: one for each loci category (IGRs, HKs, HPs and CEPs) and a global concatenated sequence involving all loci (approximately 50,000 bp for each taxon).

### Polymorphism significance

We used data from the fully sequenced genomes A/Har13 and D/UW3 for this evaluation. Thus, considering the 3,354 SNPs identified between these two genomes [23], we evaluated whether 1,099 SNPs restricted to the 51,074 bp analyzed in this study are overrepresented relative to the 2,255 SNPs found in the rest of the chromosome. We framed this as a contingency table (Table 4) with a restricted sequence of 1,519,042 bp for each strain (corresponding to the length of the D/UW3 chromosome), and we estimated *P*-values using the Fisher's exact test as well as the odds ratios with a 95% confidence interval.

**Table 4:** Contingency table for estimating polymorphism significance

	Inside region	Outside region
SNPs	1,099	2,255
Without SNPs	49,975	1,465,713

### Genomic analysis

For all individual loci and concatenated sequences, alignments of all strains were generated using LaserGene (DNASTAR, Madison, WI, USA) and MEGA 3.1 [51]. MEGA 3.1 was also used to create matrices of pairwise comparisons and to estimate the number of variable sites, the number of parsimony informative sites and overall mean genetic distances. The pairwise-deletion option was chosen to remove all sites containing missing data or alignment gaps from all distance estimations, only when the need arose and not prior to the analysis.

In order to search for distinct regions that may be associated with strains belonging to a specific disease group, SimPlot 3.5.1 [52] was used on all 51 loci. For each similarity plot, serovars were grouped according to the cell-type/organ that they infect, and nucleotide pairwise distances were calculated using the K2P method (gaps excluded; ts/tv of 2.0) in a sliding window size of 160 bp moved across the alignment in a step size of 10 bp. Additionally, for all loci where serovars E and F clustered apart from the other epithelial-genital serovars was observed, a SimPlot analysis was also performed to evaluate if the E/F nucleotide differences compared to the other genital serovars were clustered in specific domains of each locus.

To evaluate the existence of foreign genetic material [53], SWAAP 1.0.2 [54] was used to calculate the percentage of GC content for all sequences of each taxon.

### Phylogenetic analysis

Prior to the phylogenetic reconstructions, and in order to select the appropriate evolutionary models, we evaluated the homogeneity of substitution patterns between sequences by calculating the Monte Carlo test-based Disparity Index per site [55]. This gives the probability of rejecting the null hypothesis that sequences have evolved with the same pattern of substitution. The NJ method [56] was used with K2P [57], Jukes-Cantor [58] and Tamura-Nei [59] models to generate phylogenies. For the concatenated sequences, in order to examine the accuracy of the major conclusions reached from the NJ analysis, trees were also constructed under the maximum parsimony criterion [60], using the max-min branch-and-bound algorithm.

Considering that recombination disturbs a phylogenetic signal since the two parts of the recombined region may have different evolutionary histories [61,62], one locus (*ompA*) was excluded from the phylogenetic concatenated analysis, as its highly recombinant nature has already been demonstrated [48,63]. The use of outgroup sequences was discarded in the present study because no rooted trees were needed to achieve the objectives defined above. Also, the most suitable strain for use as an outgroup, *C. muridarum *(MoPn strain), has several loci that vary greatly in size and diverge from those in the *C. trachomatis *strains, which would entail the removal of a huge portion of the sequences being analyzed.

### Evolutionary γ-distance

We used a non-phylogenetic method for estimating the evolutionary distance between each pair of homologous DNA sequences, which is given by the parameter γ [34]:

γ = -2log_e_*P*

where

*P *= *n*_*xy*_/√*n*_*x*_*n*_*y*_

where *n*_*xy *_is the number of nucleotides shared by the two sequences, and *n*_*x *_and *n*_*y *_are the number of nucleotides of each sequence. For comparative purposes, we used the same set of loci as for the phylogenetic concatenated analyses, that is, we excluded the recombinant *ompA *gene. The γ variability was estimated by Monte Carlo using the alignments of each individual locus through the statistical platform R 2.5.1 [64]. Each time, 20 loci were randomly selected with replacement and γ-distances were calculated by repeating this procedure 50 times.

### Statistical analysis

The statistical association between genetic and phylogenetic variables was performed using the ANOVA test by comparing groups' population means. We considered as genetic variables the overall mean values of percent GC content, p-distance and absolute SNPs obtained for each of the selected loci. The phylogenetic variables were: clustering of strains according to tropism properties; co-segregation of E/F strains; segregation of a LGV cluster or an ocular cluster; and the 'weight' of each locus in the final concatenated tree. The homogeneity of variances was tested using the Levene's test. Whenever the hypothesis of homogeneity of variances was rejected, the non-parametric Mann-Whitney test was used to compare distributions among groups. A *P*-value of 0.05 or less was considered significant.

## Abbreviations

CEP: cell envelope protein gene; HGT: horizontal gene transfer; HK: housekeeping gene; HP: hypothetical or unclassified protein gene; IGR: intergenomic region; K2P: Kimura 2-parameter; LGV: lymphogranuloma venereum; NJ: neighbor-joining; SE: standard error; SNP: single nucleotide polymorphism.

## Authors' contributions

AN performed the experimental work, analyzed and interpreted the data, performed the statistical analysis, and wrote the manuscript. PJN performed the statistical analysis. MJB contributed to the experimental work. JPG designed the project, obtained the funding, interpreted the data, and wrote the manuscript.

## Additional data files

The following additional data are available with the online version of this paper. Additional data file 1 is a figure showing the overall mean genetic distances among all 15 *C. trachomatis *serovars for the 51 loci. Additional data file 2 is a table listing the cellular roles of the 51 loci. Additional data file 3 is a figure showing *C. trachomatis*'s evolutionary history by loci category. Additional data file 4 is a table listing the indel events found among the 15 *C. trachomatis *serovars for all loci. Additional data file 5 is a table listing the primers used for PCR and sequencing of selected loci.

## Supplementary Material

Additional data file 1 Overall mean genetic distances among all 15 *C. trachomatis *serovars for the 51 loci. Click here for file

Additional data file 2 Cellular roles of the 51 loci. Click here for file

Additional data file 3 *C. trachomatis*'s evolutionary history by loci category. Click here for file

Additional data file 4 Indel events found among the 15 *C. trachomatis *serovars for all loci. Click here for file

Additional data file 5 Primers used for PCR and sequencing of selected loci. Click here for file
