# Supplementary figures and images for: Chlamydia trachomatis diversity viewed as a tissue-specific coevolutionary arms race
Source: Genome Biol. 2008 Oct 23;9(10):R153. doi: 10.1186/gb-2008-9-10-r153 (PMC2760880; doi:10.1186/gb-2008-9-10-r153)

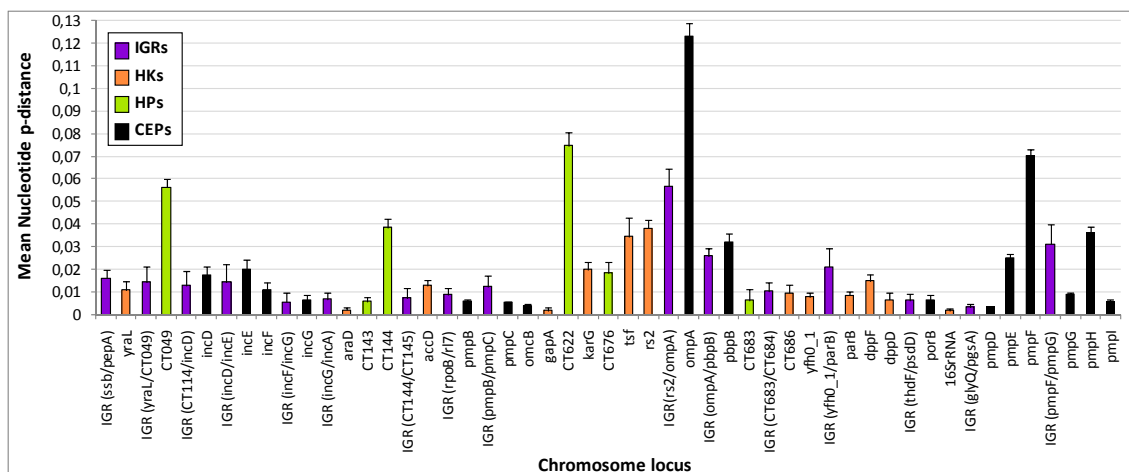

Figure S1

Supplement: Additional data file 1 — Overall mean genetic distances among all 15 C. trachomatis serovars for the 51 loci. [file gb-2008-9-10-r153-S1.pdf]

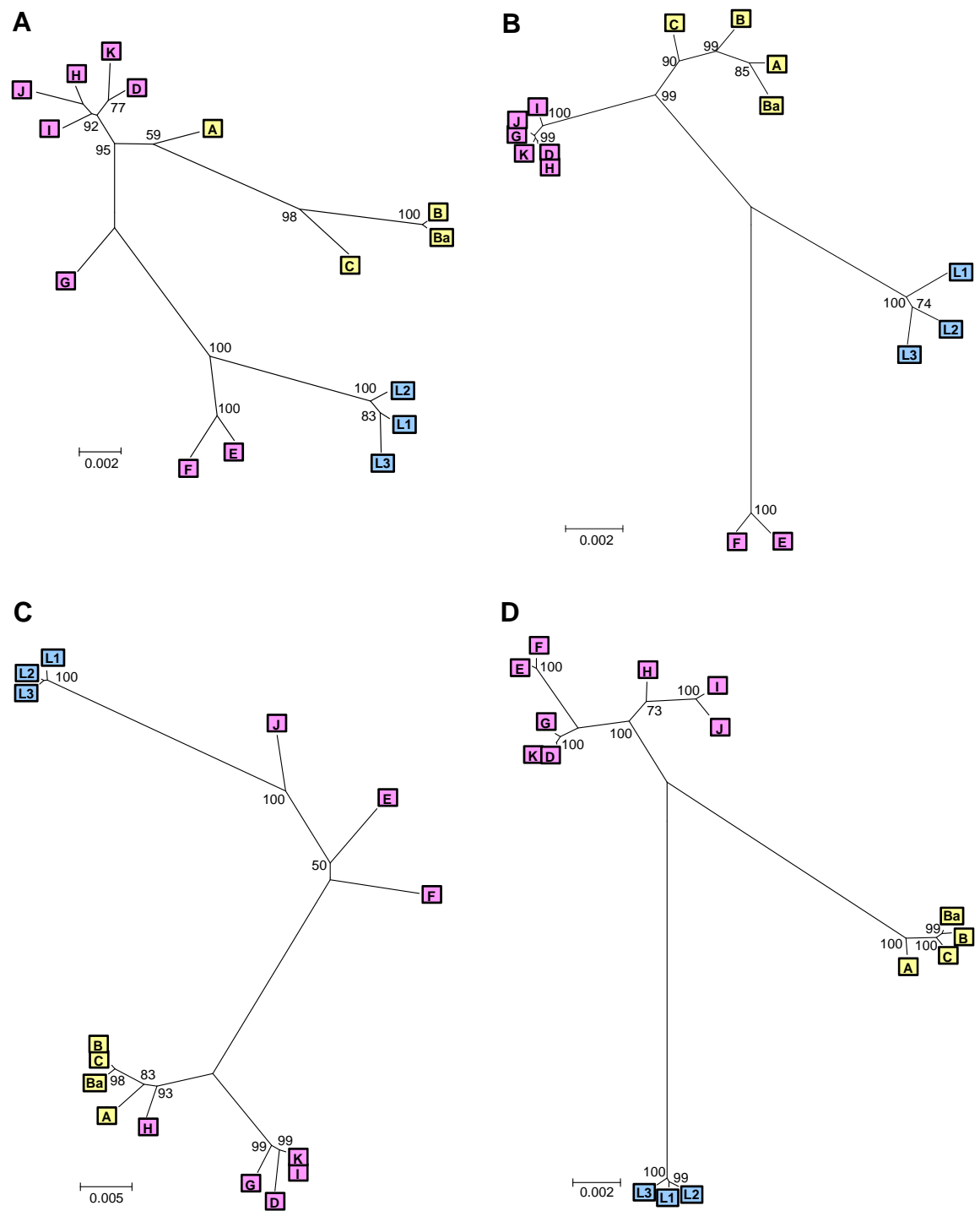

Figure S2

Supplement: Additional data file 3 — C. trachomatis's evolutionary history by loci category. [file gb-2008-9-10-r153-S3.pdf]
