# Supplementary material for: Chlamydia trachomatis diversity viewed as a tissue-specific coevolutionary arms race
Source: Genome Biol. 2008 Oct 23;9(10):R153. doi: 10.1186/gb-2008-9-10-r153 (PMC2760880; doi:10.1186/gb-2008-9-10-r153)
Supplement: Additional data file 2 — Cellular roles of the 51 loci. [file gb-2008-9-10-r153-S2.pdf]

Supplementary Table S1. Cellular role of the 51 loci

Loci	**Designation**	**Cellular Role**
		**HKs**
yraL	SAM-dependent methyltransferase	protein synthesis: tRNA and rRNA base modification
araD	Ribulose-P epimerase	energy metabolism
accD	AcCoA carboxylase/Carboxyl transferase beta	fatty acid and phospholipid metabolism
gapA	Glyceraldehyde-3-P dehydrogenase	energy metabolism
karG	Arginine kinase	regulatory function
tsf	Translation and elongation factor TS	protein synthesis
rs2	S2 ribosomal protein	protein synthesis
CT686	ABC transporter-associated protein	biosynthesis of cofactors, prosthetic groups and carriers
yfh0_1 *****	NifS-related enzyme	central intermediary metabolism: cysteine desulfurase
parB	Chromosome partitioning protein	cell process: cell division
dppF	ABC transport ATPase	transport and binding protein
dppD	ABC Transport ATPase	transport and binding protein
16SrRNA	16S ribosomal RNA	rRNA synthesis
		**HPs**
CT049	hypothetical protein	biological process and molecular function unknown
CT143	hypothetical protein	biological process and molecular function unknown
CT144	hypothetical protein	biological process and molecular function unknown
CT676	ClpC ATPase	unknown function
CT683	TPR-motif protein	unknown function
CT622	CHLPN 76kDa protein-like	unclassified: role not assigned
		**CEPs**
incD	Inclusion membrane protein D	cell envelope
incE	Inclusion membrane protein E	cell envelope
incF	Inclusion membrane protein F	cell envelope
incG	Inclusion membrane protein G	cell envelope
pmpB	Polymorphic membrane protein B	cell envelope
pmpC	Polymorphic membrane protein C	cell envelope
omcB	60kDa cysteine-rich outer membrane protein	cell envelope
ompA	Major outer membrane protein	cell envelope
pbpB	PBP2-transglycolase/transpeptidase	cell envelope: biosynthesis and degradation of murein sacculus and peptidoglycan
porB	Outer membrane protein B	cell envelope
pmpD	Polymorphic membrane protein D	cell envelope
pmpE	Polymorphic membrane protein E	cell envelope
pmpF	Polymorphic membrane protein F	cell envelope
pmpG	Polymorphic membrane protein G	cell envelope
pmpH	Polymorphic membrane protein H	cell envelope
pmpI	Polymorphic membrane protein I	cell envelope
		**IGRs**
ssb/*pepA*	IGR between ssDNA binding protein and Leucyl	

	aminopeptidase A genes
yraL/CT049	IGR between *yraL* and CT049 genes
CT114/*incD*	IGR between hypothetical protein CT114 and *incD* genes
incD/*incE*	IGR between *incD* and *incE* genes
incF/*incG*	IGR between *incF* and *incG* genes
incG/*incA*	IGR between *incG* and *incA* genes
CT144/CT145	IGR between CT144 and hypothetical protein CT145 genes
rpoB/*rl7*	IGR between RNA polymerase beta and L7 ribosomal protein genes
pmpB/*pmpC*	IGR between *pmpB* and *pmpC* genes
rs2/*ompA*	IGR between *rs2* and *ompA* genes
ompA/*pbpB*	IGR between *ompA* and *pbpB* genes
CT683/CT684	
yfh0_1/*parB*	IGR between *yfh0_1* and *parB* genes
thdF/*psdD*	IGR between Thiophene/Furan oxidation protein and Phosphatidylserine decarboxylase genes
glyQ/*pgsA*	IGR between Glycyl tRNA synthetase and CDP-diacylglycerol-glycerol-3-P 3-phosphatidyltransferase genes
pmpF/*pmpG*	IGR between *pmpF* and *pmpG* genes

Gene names and ORF numbers are based on the *C. trachomatis* D/UW3 genome annotation (GenBank No. AE001273). Cellular roles are designated according to TIGR annotation [65].

***** The sequence of this gene is 100% similar to the one of *yfh0_2* for the *C. trachomatis* A/Har13 strain (GenBank No. CP000051), which points to an annotation incongruence among the two strains.
