# Supplementary material for: Chlamydia trachomatis diversity viewed as a tissue-specific coevolutionary arms race
Source: Genome Biol. 2008 Oct 23;9(10):R153. doi: 10.1186/gb-2008-9-10-r153 (PMC2760880; doi:10.1186/gb-2008-9-10-r153)
Supplement: Additional data file 4 — Indel events found among the 15 C. trachomatis serovars for all loci. [file gb-2008-9-10-r153-S4.pdf]

Supplementary Table S2. Deletion events among the 15 *C. trachomatis* reference strains

Loci	Deletion events		
	Position (bp)	Size (bp)	Reference strains
IGR (*ssb*/*pepA*)	29-30	2	All but LGV
yraL	---	---	---
IGR (*yraL*/CT049)	62	1	All but H and J
CT049	64-90	27	All but H and J
	172-177	6	All but F, H, I and J
	379-387	9	H, I and J
	595-597	3	All but H, I and J
	883-894	12	H, I and J
	1228-1233	6	D, G, H and K
	1462-1467	6	All but D, G, H and K
IGR (CT114-*incD*)	22	1	A-C
	28	1	E
	86	1	LGV
	89-131	43	A-C
	121	1	LGV
incD	1-28	28	All but A-C
	36	1	A-C
	37-39	3	All but A-C
	437	1	LGV
	458-473	16	All but LGV
IGR (*incD*/*incE*)	1-16	16	LGV
incE	---	---	---
incF	---	---	---
IGR (*incF*/*incG*)	---	---	---
incG	---	---	---
IGR (*incG*/*incA*)	55-57	3	A, H, J, L1 and L3
	56-57	2	B-C, D, F, G, I, K and L2
	122	1	D, G, I and K
araD	---	---	---
CT143	---	---	---
CT144	289-297	9	All but J and LGV
IGR (CT144/CT145)	---	---	---
accD	---	---	---
IGR (*rpoB*/*rl7*)	---	---	---
pmpB	1158-1163	6	A-D, G-K
	1161-1163	3	LGV
	1971-1973	3	E and F
	2844-2852	9	LGV
IGR (*pmpB*/*pmpC*)	98	1	LGV
	118	1	A-C and L2
pmpC [a]	296-337	42	D, G-K
	2365-2394	30	LGV

omcB	---	---	---
gapA	---	---	---
CT622	244-249	6	LGV
	253-270	18	D, I and K
	262-267	6	A-C, G and H
karG	---	---	---
CT676	---	---	---
tsf	---	---	---
rs2	733-744	12	B
	799-781	3	C
	799-807	9	All but B and C
IGR (*rs2*/*ompA*)	17	1	F and L1
	72	1	E, F and LGV
	83-84	2	A-C, D, G-K
	221-222	2	C
	222	1	A, D, H-K
	233	1	G
	238	1	A, C, D, H-K
	255	1	G
	257	1	H and J
	259-262	4	A, C, D, H-K
	272-273	2	A, C, D, H-K
	286-287	2	A, C, D, H-K and L3
	287	1	B, Ba, E, L1 and L2
	329-337	9	All but LGV
	364-369	6	B, Ba, E-G, LGV
	391	1	A, C, D, H-K and L3
IGR (*ompA*/*pbpB*)	493-513	21	B, Ba and F
pbpB	---	---	---
CT683	---	---	---
IGR (CT683/CT684)	204-205	2	E, F and LGV
	205	1	A-C, D, G, I-K
	227	1	All but J
CT686	---	---	---
yfh0_1	---	---	---
IGR (*yfh0_1*/*parB*)	---	---	---
parB	---	---	---
dppF	---	---	---
dppD	---	---	---
IGR (*thdF*/*psdD*)	126	1	All but LGV
porB	---	---	---
16SrRNA	835	1	All but A
	1024	1	A
IGR (*glyQ*/*pgsA*)	170	1	D and K
	310-317	8	G and LGV

pmpD	3124-3126	3	E, F and LGV
pmpE	355-357	3	D-H and K
	1048-1053	6	A-C
	1159-1161	3	A-C, I and J
	1411-1419	9	A-C
	1426-1428	3	A-C
	1540-1551	12	All but A-C
pmpF	1292-1294	3	LGV
	2104-2106	3	H, I and J
	2115-2117	3	LGV
IGR (*pmpF*/*pmpG*)	28	1	D-G, K and LGV
	74	1	D-G, K and LGV
pmpG	1046-1048	3	L2
pmpH	484-519	36	LGV
	484-489	6	I and J
	691-693	3	LGV
	700-708	9	All but LGV
	868-870	3	L1 and L3
	886-888	3	All but A-C
	1675-1677	3	A-C
	1885-1890	6	All but A-C
pmpI	---	---	---

[a] For *pmpC* the information presented in this table refers to remnant fragments of the putative IS-like elements (see ref. 28 in the main text).
