# Supplementary material for: Chlamydia trachomatis diversity viewed as a tissue-specific coevolutionary arms race
Source: Genome Biol. 2008 Oct 23;9(10):R153. doi: 10.1186/gb-2008-9-10-r153 (PMC2760880; doi:10.1186/gb-2008-9-10-r153)
Supplement: Additional data file 5 — Primers used for PCR and sequencing of selected loci. [file gb-2008-9-10-r153-S5.pdf]

Table S3. Primers used for PCR and sequencing

Locus	Primers	Primer sequence (5´ to 3´)	Amplicon size (bp)
IGR (*ssb*/*pepA*)	IGR 44/45-1 [a]<br>IGR 44/45-2 [a]	TTCCGGATCTTCTCGTCCTGATG<br>GCACCCCTGCTGCCAAGTTC	915
IGR (CT114/*incD*), *incD*, IGR (*incD*/*incE*), *incE*, *incF*, IGR (*incF*/*incG*), *incG*, and IGR (*incG*/*incA*)	inc-1 [a]<br>inc-2 [a]<br>inc-4 [a]<br>inc-5 [a]	AGCTCAGCGAGGGCTTGAAGA<br>GTTCGCCGATTAGCGCATAACC<br>GCTTCATTGAGAGGCTGTT<br>CGCCGCTGATAATATGGATA	2387
CT143, CT144, and IGR (CT144/CT145)	CT144-1 [a]<br>CT144-2 [a]<br>CT144-3 [a]	CTGCGCGACCCACCGTAAT<br>TCAACTGCGGCTCGCTCTTC<br>ATAGCGCTGAACGCATACTT	1531
IGR (*rpoB/rl7*)	IGR 315/316-1 [a]<br>IGR 315/316-2 [a]	TTCCCCGACGAATACACTCTTC<br>ATCGGGGTTCTGAAAGTTGTTAGA	799
CT686, *yfh0_1*, and IGR (*yfh0_1*/*parB*)	yfh0-1 [a]<br>yfh0-2 [a]<br>yfh0-3 [a]	TCCTCGCGGGACGCTATCTA<br>TCCCCATTACGGATCTCCCTAAC<br>GCACCATGCTAATCTCGTAT	1795
parB	parB-1 [a]<br>parB-2 [a]	TGCGGAGTGGGCATCTCTTGATTT<br>CTGGGGGACAGCTACAACGCATCT	1308
dppF, and *dppD*	dppF-1 [a]<br>dppF-2 [a]	AACGGTAAAGCCTCAGGGAAGACG<br>TGTATGCAGGCCGTATGGCAGAAT	1210
porB	porB-1 [a]<br>porB-2 [a]	TGGGAAGGCGGTAGTATTGTTTCA<br>ATGCCGATTACCACCAGCGTCTAT	712

Primers were designed based on published genome sequences of reference strains A/Har13 and D/UW3 (GenBank CP000051 and AE001273, respectively).

[a] Primers also used for automated sequencing.
